# Is chemical exposure present in informal work associated with Sars-CoV-2 infection?

**DOI:** 10.11606/s1518-8787.2023057004829

**Published:** 2023-05-11

**Authors:** Fernanda Junqueira Salles, Isabelle Nogueira Leroux, Alda Neis Miranda de Araujo, Nilson Antonio Assunção, Maciel Santos Luz, Glaucia Raquel Luciano da Veiga, Fernando Luiz Affonso Fonseca, Fredi Alexander Diaz-Quijano, Kelly Polido Kaneshiro Olympio

**Affiliations:** I Universidade de São Paulo Faculdade de Saúde Pública Departamento de Saúde Ambiental São Paulo SP Brasil Universidade de São Paulo. Faculdade de Saúde Pública. Departamento de Saúde Ambiental. São Paulo, SP, Brasil; II Universidade Federal de São Paulo Instituto de Ciências Ambientais, Químicas e Farmacêuticas Diadema SP Brasil Universidade Federal de São Paulo. Instituto de Ciências Ambientais, Químicas e Farmacêuticas. Diadema, SP, Brasil; III Instituto de Pesquisas Tecnológicas de São Paulo Laboratório de Processos Metalúrgicos São Paulo SP Brasil Instituto de Pesquisas Tecnológicas de São Paulo. Laboratório de Processos Metalúrgicos. São Paulo, SP, Brasil; IV Centro Universitário Faculdade de Medicina do ABC Laboratório Clínico Santo André SP Brasil Centro Universitário Faculdade de Medicina do ABC. Laboratório Clínico. Santo André, SP, Brasil; V Universidade de São Paulo Faculdade de Saúde Pública Departamento de Epidemiologia São Paulo SP Brasil Universidade de São Paulo. Faculdade de Saúde Pública. Departamento de Epidemiologia. São Paulo, SP, Brasil

**Keywords:** Chemical Compound Exposure, Occupational Exposure, Informal Sector, Physical Distancing, COVID-19

## Abstract

**OBJECTIVE:**

To compare the incidence of covid-19 symptoms between informal home-based workers and a control group and to assess the association of these cases with blood elements concentrations and other relevant risk factors for Sars-Cov-2 infection.

**METHODS:**

Welders chemically exposed to potentially toxic elements (PTEs) (n = 26) and control participants (n = 25) answered questionnaires on adherence to social distancing and signs and symptoms of the disease for five months during the covid-19 pandemic. After follow-up, covid-19 serology tests were performed on a subsample of 12 chemically exposed workers and 20 control participants. Before the pandemic, PTE concentrations in blood (As, Mn, Ni, Cd, Hg, Sb, Sn, Cu, Zn, and Pb) were measured by ICP-MS.

**RESULTS:**

The chemically exposed group had higher lead and cadmium levels in blood (p < 0.01). The control group presented lower adherence to social distancing (p = 0.016). Although not significant, welders had a 74% greater chance of having at least one covid-19 symptom compared with control participants, but their adherence to social distancing decreased this chance by 20%. The use of taxis for transportation was a risk factor significantly associated with covid-19 symptoms.

**CONCLUSION:**

The lower adherence to social distancing among the control group greatly influences the development of covid-19. The literature lacks data linking exposure to PTEs and Sars-Cov-2 infection and/or severity. In this study, despite chemical exposure, working from home may have protected welders against covid-19, considering that they maintained greater social distancing than control participants.

## INTRODUCTION

Exposure to potentially toxic elements (PTEs) occurs in many occupational activities. Welding materials can include metal alloys, mixtures made of different steels and chemical elements, which can be inhaled in the form of welding fumes. Thus, welding is one of the occupations most associated with respiratory diseases^1,2^. Contamination by PTEs can cause other adverse effects to health and the environment. This risk is even higher when occupational activities are informal and performed at home, where families may be at risk of occupational exposure in the absence of occupational hygiene^3^.

Exposure to pollutants, overcrowded housing, and socioeconomic stress are conditions that may promote greater susceptibility and the development of more severe symptoms of infectious diseases^4^, including covid-19, which is caused by the new coronavirus. In 2020, one in six people infected with covid-19 become seriously ill and experience breathing difficulties. Older adults and individuals with underlying health conditions, such as high blood pressure, heart and lung problems, diabetes, or cancer, are at increased risk of becoming seriously ill^5^.

Informal, home-based, and outsourced welders are influenced by their internal exposome and studies on associations with diseases should consider this type of occupational activity^6^. In 2019, a proteomic study based on the saliva of informal and home-based jewelry welders showed that exposure to PTEs (As, Cr, Cd, Cu, Mn, Ni, Pb, and Zn) modulates protein expression. According to the results, welders may have immunity problems and their genes were overrepresented in metabolic processes, catalytic activity, stimulus-response, response to toxic substances, metabolism activity, apoptosis, changes in the cell cycle, and stress response^7,8^.

The global scenario of the covid-19 pandemic has increased the importance of assessing this type of occupational exposure and the negative health effects related to infectious diseases. Thus, this study aimed to compare the incidence of covid-19 symptoms between home-based and informal workers engaged in jewelry and fashion jewelry production and a control group, assessing the association of symptomatic episodes with blood metal concentrations and other relevant risk factors for Sars-Cov-2 infection.

## METHODS

### Study Population

This longitudinal study included home-based and informal jewelry welders of a local productive arrangement in the city of Limeira, São Paulo, and a control group with individuals who lived in the same neighborhood as the workers, but were not engaged in an occupational activity associated with chemical exposure.

The local productive arrangement is an important jewelry and fashion jewelry production center in Brazil, responsible for 60% of the country’s jewelry production^9^. Previous studies published by the research group present further details on the work processes and characteristics of informal home-based activities^10-13^.

All adults participating in a preliminary study (*A era “omics” voltada para a sociedade: o impacto do trabalho formal e informal sobre o expossoma dos trabalhadores com ênfase em metabolômica, transcriptômica e lipidômica*) were invited to participate in this study. The initial recruitment was performed with the help of community agents of family health centers. All participants were women, in order to standardize this variable. Participants in the exposed group performed jewelry welding at home. Participants in the control group lived in the same neighborhood as the workers (at least four houses away) and performed no activity with chemical exposure. Usually, they worked outside the home. Participants who met this inclusion criterion, agreed to participate in the study, and signed the informed consent form were included. Women younger than 18 years and who were pregnant or diagnosed with cancer were excluded.

Welders occupationally and chemically exposed to PTEs (n = 26) and control participants (n = 25) were followed up for five months during the covid-19 pandemic (June to September and December 2020) for the characterization of symptoms and preventive behaviors. With a sample size of 51 participants, ratio differences of 40% with a power of 80% could be found for dichotomic variables, as well as differences in means that exceed 1.2 times the standard deviation between samples with a power of 85% for quantitative variables (EpiData, version 3.11). At the end of the follow-up, covid-19 serology tests were performed on a subsample of 32 participants who agreed to undergo the test, including 12 from the chemically exposed group and 20 from the control group (Figure).

**Figure:**
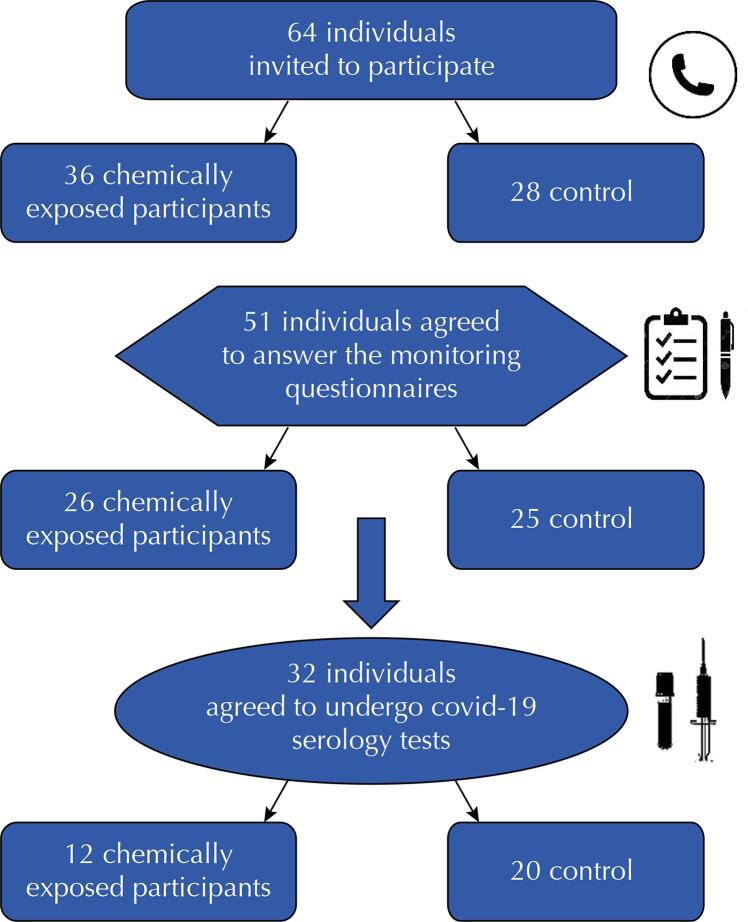
Flowchart of participant adherence throughout the study.

### Determination of Metals in Blood

Before the pandemic, PTE concentrations in the blood of participants (As, Mn, Ni, Cd, Hg, Sb, Sn, Cu, Zn, and Pb) were measured using an inductively coupled plasma mass spectrometer (ICP-MS Thermo, iCAP TQ ICP-MS, Bremen, Germany). Blood samples (6 mL) were collected in October 2019 at the participants’ home and stored in heparin trace element-free tubes (Vacutainer^®^). Samples were diluted 1:50 in a 15 mL Falcon^®^ polypropylene tube (Becton Dickinson) with a solution of 0.01% (v v^1^) Triton^®^ X-100, 0.5% (v v^1^) nitric acid, and 1 mL of an internal standard solution of Y, Ga, Ir, and Tb. High purity deionized water (resistivity: 18.2 M Ω cm at 25°C) was used to prepare samples and solutions. Quality control of analytical results was performed by calibration curves, blank analysis, and an analysis of the certified reference material (Seronorm^®^ TE Whole Blood Level II, Stasjonsveien) for every 15 samples. A deviation between replicates (RSD) of 30% was considered.

### Self-reported covid-19 Monitoring Questionnaire

During the pandemic, all participants answered questionnaires via Google Forms every 30 days for five months to provide information on adherence to social distancing (days and hours away from home per week and the use of public transportation) and signs and symptoms of the disease (cough, congestion, sore throat, headache, conjunctivitis, diarrhea, loss of taste or smell, shortness of breath, and fever). Participants also answered questions on their current working conditions and the effect of the pandemic on their jobs (continuity, decrease, or interruption of activities). Thus, information included all symptoms perceived from October 2020 to the day of serology tests (December 2020). Throughout the five-month follow-up, 197 observations were collected from the 51 participants and 69 symptomatic episodes were identified.

### Serology Tests

The material for blood collection was prepared at the Laboratory for the Analysis of Human Exposure to Environmental Contaminants of the School of Public Health of the Universidade de São Paulo, São Paulo, Brazil. Blood collection was performed by an experienced nurse at the participants’ home by prior appointment. Venous blood was collected in BD Vacutainer^®^ EDTA tubes.

After blood collection, samples were labeled and transported in an ice box to the laboratory of the Faculdade de Medicina do ABC, where antibody serology tests were performed. First, samples were centrifuged to separate the serum. Then, 10 μL of serum was diluted to 1.0 mL in the sample buffer and mixed thoroughly using a vortex. Analyses were performed for total antibodies and IgG, which was detected using Euroimmun Elisa tests (Euroimmun US, Inc., NJ, USA). In these tests, the antigen is the structural recombinant protein, the S1 subunit of the SARS-CoV-2 spike protein, which is more specific than other antigens. The Advia Centaur^®^ SARS-CoV-2 Total (COV2T) assay (Siemens Healthcare Diagnostics Inc., NY, USA) was used for *in vitro* diagnostics in the qualitative detection of total antibodies (including IgG and IgM) to help with the diagnosis of patients with suspected SARS-CoV-2 infection and the identification of patients with an adaptive immune response, pointing to a recent or previous infection.

### Statistical Analysis

The chemically exposed and control groups were compared using chi-square tests for dichotomous variables (adherence to social distancing, days away from home per week, hours spent away from home when going out, use of public transportation, and positive or negative results of serology tests). Moreover, the t-test (parametric) was used to compare continuous variables (age and Mg, Ni, Zn, and Sn concentrations in blood). The Mann–Whitney test (nonparametric) was used for body mass index (BMI) and Cr, Co, As, Cd, Hg, and Pb concentrations in blood. The frequency of symptoms (cough, congestion, sore throat, loss of taste or smell, shortness of breath, and fever) and the difference between symptoms in the groups were assessed (Fisher’s exact test or chi-square test).

Participants’ symptoms were also analyzed by assessing the incidence of a composite outcome defined for the presence of at least one covid-19 symptom (cough, congestion, sore throat, loss of taste or smell, shortness of breath, and fever) during repeated assessments. Later, the associations between this composite outcome and variables, such as social distancing parameters and PTE levels, were assessed using random-effect logistic regression, considering the participant as the clustering variable. Statistical analysis was performed using Stata 14.2.

## RESULTS

Table 1 presents PTE levels in the blood of participants, their age, and BMI, which are considered risk factors for Sars-Cov-2 infection. BMI was statistically higher in the control group compared with the chemically exposed group (p = 0.01) and lead (Pb) and cadmium (Cd) levels in blood were higher in the chemically exposed group (p = 0.01 and p < 0.001, respectively).

**Table 1:** Age, body mass index (BMI), and potentially toxic element (PTE) distribution by group in Limeira, SP, Brazil.

Variable	Chemically exposed		Control		Total		p-value
		
	Mean	SD	Mean	SD	Mean	SD	
Age (years old)	37.23	11.54	35.32	9.19	36.29	10.40	0.52
BMI (kg/m^2^)	27.89	7.47	30.76	6.41	29.53	7.01	0.01
Chromium (µg L^−1^)	1.22	0.81	1.50	1.64	1.36	1.28	0.58
Manganese (µg L^−1^)	8.98	3.57	8.12	2.58	8.56	3.12	0.33
Nickel (µg L^−1^)	3.31	1.64	3.13	1.71	3.22	1.65	0.73
Copper (µg L^−1^)	1,119.77	230.00	1,174.40	210.71	1,146.55	220.28	0.37
Zinc (µg L^−1^)	4,360.99	1,118.09	4492.29	1,133.27	4,425.35	1,116.21	0.68
Arsenic (µg L^−1^)	0.44	0.22	0.97	2.11	0.70	1.49	0.42
Cadmium (µg L^−1^)	1.13	1.55	0.15	0.35	0.66	1.24	< 0.001
Tin (µg L^−1^)	0.97	0.26	0.95	0.34	0.96	0.30	0.83
Mercury (µg L^−1^)	1.24	0.57	1.16	0.94	1.20	0.77	0.16
Lead (µg dL^−1^)	2.15	1.53	1.09	0.51	1.63	1.26	0.01

Headache, congestion, and cough were the most frequent symptoms (45.2%, 19.3%, and 15%, respectively). Congestion was the only symptom that statistically differed between groups (p = 0.02), as it was more frequently among welders. Other symptoms had borderline significance (p = 0.05) at different points during follow-up: cough and diarrhea were more prevalent among welders while control participants reported more tiredness. Table 2 shows the prevalence of adherence to covid-19 preventive behaviors and symptomatic episodes in the exposed group.

**Table 2:** Prevalence of social distancing, symptoms, and covid-19 during the five-month follow-up (197 observations) by group in Limeira, SP, Brazil.

Variable	Chemically exposed		Control		Total		p-value
		
	n = 86	43.6%	n = 111	56.4%	n = 197	100%	
Presence of covid-19 symptoms							0.098
Yes	36	41.86%	33	29.73%	69	35.03%	
No	50	58.14%	78	70.27%	128	64.97%	
Adherence to social distancing							0.018
Yes	73	84.88%	78	70.27%	151	76.65%	
No	13	15.12%	33	29.73%	46	23.35%	
Days away from home per week							< 0.0001
≤ 4 days	77	89.53%	67	60.53%	144	73.10%	
> 4 days	9	10.47%	44	39.64%	53	26.90%	
Hours spent away from home when going out							< 0.0001
≤ 4 hours	76	88.37%	73	65.77%	149	75.63%	
> 4 hours	10	11.63%	38	34.23%	48	24.37%	
Use of public transportation							
Bus (n = 17)	2	11.76%	15	88.24%	17	100%	0.005
Car via transport apps (n = 44)	23	52.27%	21	47.73%	44	100%	0.228
Taxi (n = 8)	3	37.50%	5	62.50%	8	100%	1.000
Serology test (n = 32)							0.139
Positive (n = 11)	2	18.18%	9	81.82%	11	100%	
Negative (n = 21)	10	45.45%	11	54.38%	22	100%	

The control group presented lower adherence to social distancing than the chemically exposed group (p = 0.02), corroborating the greater number of days and hours spent away from home (p < 0.0001) and the higher use of public transportation, such as the bus, by control participants (p = 0.006) compared with chemically exposed participants. This result is in line with participants’ occupational activities, since welders performed informal home-based work whereas control participants needed to leave home for work several days per week and stay out for longer periods.

The covid-19 pandemic affected the occupational activity of the chemically exposed group, which decreased or was interrupted more frequently than the activities of the control group (p < 0.001). During follow-up, 41.4% of informal welders reported a reduction in the amount of work and 31% stopped working during the pandemic. For the control group, work conditions were quite different: for 36% of control participants, the pandemic did not affect their work, and 39% were not working before the pandemic.

In total, 63% of participants agreed to undergo covid-19 serology tests (n = 32), especially control participants (n = 20). Among participants who refused to undergo the test, 73.7% were from the chemically exposed group (p = 0.012) and most of them did not present the composite outcome of covid-19 symptoms (74.1%; p = 0.04; Table 3).

**Table 3:** Observations considering the presence of covid-19 symptoms throughout the follow-up, according to participants’ acceptance of being tested for covid-19 and groups.

Accepted being tested for covid-19	Observations with the presence of Covid-19 symptoms						

	Yes		No		Total		p-value
		
	n	%	n	%	n	%	
Chemically exposed group							0.043
Yes	29	49.15	30	50.85	59	100	
No	7	25.93	20	74.07	27	100	
Control group							0.104
Yes	27	27.27	72	72.73	99	100	
No	6	50.00	6	50.00	12	100	
Total							0.805
Yes	56	35.44	102	64.56	158	100	
No	13	33.33	26	66.67	39	100	

We observed no significant association between test results and covid-19 symptoms, as presented by the composite outcome (p = 0.96). The results of serology tests also did not differ statistically between the two groups (p = 0.102). PTE levels were also similar for both positive and negative test results, except for Pb (p = 0.0306), as its levels were higher in participants who tested negative (1.87 ± 1.32 µg dL^1^) compared with those who tested positive (0.99 ± 0.53 µg dL^1^).

We analyzed associations between covid-19 symptoms and risk factors or preventive behaviors using random-effect logistic regression, considering the participant as the clustering variable. Although not significant, chemically exposed participants had a 74% higher chance of having at least one covid-19 symptom compared with the control group, however, adherence to social distancing decreased these chances by 20%. The use of taxis for transportation was significantly associated with symptoms, as the odds ratio of the composite outcome for users was 6.04 times greater than for non-users (Table 4). When adjusted for age and BMI, the odds ratio of the composite outcome for taxi users was 4.82 greater than for non-users (95%CI: 0.74–31.52; p = 0.10).

**Table 4:** Associations with 95% confidence interval (95%CI) between the composite outcome (the presence of at least one covid-19 symptom) and risk factors (n = 197 observations) in Limeira, SP, Brazil.

Risk factor	OR	95%CI	p-value
Group (chemically exposed or control)	1.74	0.83–3.68	0.14
Age (years old)	1.00	0.97–1.04	0.83
BMI (kg/m^2^)	0.99	0.93–1.06	0.85
Chromium (ppb)	0.88	0.64–1.21	0.43
Manganese (ppb)	1.08	0.95–1.22	0.26
Nickel (ppb)	1.11	0.84–1.46	0.48
Copper (ppb)	1.00	1.00–1.00	0.50
Zinc (ppb)	1.00	1.00–1.00	0.91
Arsenic (ppb)	1.01	0.75–1.35	0.96
Cadmium (ppb)	1.09	0.80–1.49	0.59
Tin (ppb)	3.65	0.84–15.84	0.08
Mercury (ppb)	0.64	0.31–1.30	0.22
Lead (µg/dL)	1.22	0.89–1.68	0.22
Adherence to social distancing	0.80	0.34–1.86	0.60
Days away from home per week	1.02	0.87–1.19	0.83
Hours spent away from home when going out	1.09	0,86–1,38	0.48
Use of public transportation	1.25	0.34–1.87	0.61
Bus use	1.95	0.59–6.44	0.28
Car use via transport apps	0.96	0.43–2.18	0.93
Taxi use	6.04	1.05–34.87	0.04

## DISCUSSION

This study shows the incidence of symptomatic Sars-Cov-2 infections during the current pandemic. We found no statistical difference concerning this outcome, but the odds ratio of having covid-19 symptoms was higher for chemically exposed participants and the number of positive serology tests was greater in the control group. Moreover, we observed a relationship between using taxis and the incidence of this outcome, suggesting that factors related to transportation represent a key contributor to the risk associated with these respiratory diseases.

The results of this study showed no significant difference in covid-19 incidence between the two groups, although the control group had a greater number of positive serology tests for the SARS-CoV-2 antibody. This group probably had more cases due to lower adherence to social distancing and the need to work away from home several days per week. Social distancing is a primary tool and an effective strategy to combat the transmission of the coronavirus^14,15^. However, evidence shows systematic differences in adherence to social distancing among income levels and lower-income communities are typically more at risk of covid-19^16^. This factor has an important influence in Brazil, considering social and regional inequalities that require urgent economic measures to guarantee a minimum income for more vulnerable populations and ensure employment protection for salaried workers^17^.

Welders are probably at higher risk of having covid-19 symptoms because they are exposed to toxic elements during their occupational activities, which can cause adverse health effects. The protein expression of welders shows that they may have immunity problems^7^. According to previous studies^11,13^, Cd and Pb levels in urine and blood may explain some immunotoxic effects. Cd is involved in the modulation of immune responses and associated with the development of abnormal inflammatory responses and cytokine and chemokine production, which may result in tissue damage, organ dysfunction, and increased susceptibility to infections^18^. The effects of Pb on the immune system are not well-established, however, studies suggest that it can affect immune response by changing T helper cell function, decreasing peripheral blood populations, and increasing susceptibility to autoimmunity and hypersensitivity^19,20^. Thus, this is a high-risk group mainly due to the uncontrolled occupational activity, which is responsible for the chemical exposure that pose a health risk to workers, their relatives, and children sharing the same household^11,21^.

Some welders already had respiratory symptoms before the pandemic, caused mainly by occupational exposure, which may explain the lack of association between covid-19 test results and symptoms. In a descriptive analysis performed in 2019, before the covid-19 pandemic^22^, the symptom of shortness of breath was statistically different between groups (p = 0.004; 88.9% of the chemically exposed group versus 11.1% of the control group). Moreover, 40.9% of the sample had bronchitis, asthma, or upper respiratory infections (sinusitis, rhinitis, otitis, pharyngitis, tonsillitis). Welders are at risk of developing various respiratory signs and symptoms, since they are exposed to fumes made of a complex mixture of toxic elements^1,23^. Studies confirmed the association between welding and reduced pulmonary function, chronic bronchitis, and increased prevalence of wheezing^2,23,24^. Based on evidence from human and animal studies, the International Agency for Research on Cancer^25^ classified welding fumes as possibly carcinogenic to humans. According to the Occupational Safety and Drug Administration, exposure to welding fumes can cause respiratory problems, cancer, ulcers, damage to the kidneys and nervous system, among other diseases^26^.

Besides its direct effect on respiratory physiology, exposure to toxic metals in welding fumes significantly modulate immune mechanisms, increasing welders’ susceptibility to respiratory infections^1,27^. The adverse effects of toxic environmental and occupational exposure deteriorate the immune system, reducing barrier function, worsening airway inflammation, oxidative stress, and apoptosis, and increasing the likelihood of comorbidities, symptom severity, and covid-19 mortality^4,28^. Data show that As, Cd, Hg, and Pb exposure is associated with respiratory dysfunction and the severity of respiratory diseases, including influenza and pneumonia^28^.

In this study, participants who tested negative for the SARS-CoV-2 antibody had the highest Pb concentrations in blood, which may be due to possible immunosuppressive effects of Pb. This element can affect humoral immune response by reducing IgA and IgG production^29,30^. Pb levels in the blood of welders are associated with a significant lack of T helper lymphocytes, IgG, IgM, C3, C4 complement levels, chemotaxis, and neutrophil migration^31^. Jusko et al.^32^ found that the odds of children seronegative to measles, mumps, and rubella increased with higher Pb concentrations in blood, which shows potential immunosuppression at Pb concentrations in blood < 5 µg dL^1^.

The only risk factor with a significant association with covid-19 symptoms was the use of taxis for transportation. Previous studies presented evidence on human-to-human transmission of covid-19 in public transport vehicles, such as trains, buses, and airplanes^33^. Public transports are confined spaces conducive for the transmission of infectious diseases caused by respiratory viruses and may play a crucial role in the nationwide spread of covid-19^33,34^. To prevent or control the spread of the disease, improving health education of passengers and drivers on the use of masks and hand hygiene and the ventilation and disinfection of vehicles is paramount^34^.

This study cannot exclude cross-reactivity with SARS-CoV-1 due to the high degree of similarity between S1 of SARS-CoV-1 and SARS-CoV-2. However, SARS-CoV-1 has not circulated since 2003 and specific antibodies were not detected until many years after infection. Thus, the presence of SARS-CoV-1 antibodies in this population is highly unlikely^35^. The performance of other tests to diagnose covid-19, the number of samples analyzed, the nutritional status of participants, and the duration of the observation period can be limitations of this study. The sample size used may produce inaccurate estimates, with wide confidence intervals. Moreover, the power of the study may be insufficient to identify all associations. However, the interpretation of odds ratios shows associations between chemical exposure and covid-19 symptoms. These results point insights raised in an exploratory study. The associations observed between transportation (taxi) and the prevalence of covid-19 symptoms reinforce other studies that found robust associations^33,36^. Although the symptom outcome may have other etiologies, acute syndromes are relevant in a pandemic context. Moreover, if we considered a non-differential lack of specificity between different levels of exposure, the association could be even greater with real covid-19 cases^37^.

## CONCLUSION

This study showed results for a population with difficult access and very peculiar characteristics due to informal work and chemical exposure. The literature lacks data linking exposure to PTEs and Sars-Cov-2 infection and/or severity. Both epidemiological and laboratory studies are required to characterize the association between this exposure and the risk of covid-19. Individual differences in immune defense, nutrient supply, age, sex, comorbidities, socioeconomic class, as well as environmental exposure and its role in susceptibility to infectious diseases, need to be further studied and understood. Despite chemical exposure, working from home may have protected welders against covid-19, considering that they maintained greater social distancing than control participants.

We found no significant association between the results of serology tests and the incidence of covid-19 symptoms between groups, although most positive tests belonged to the control group. This may be due to lower adherence to social distancing in this group, along with the greater number of days and hours spent away from home and the higher use of public transportation. The use of taxis for transportation was significantly associated with covid-19 symptoms and users had 6.04 times greater odds of the outcome composite than non-users. Therefore, lower adherence to social distancing among control participants, who need to leave home for work, is probably the major influence on the development of covid-19. However, welders had 74% greater odds of having covid-19 symptoms than the control group, although adherence to social distancing decreased these odds by 20%. Further studies are needed to deeper understand the effect of chemical exposure on Sars-Cov-2 infection.
